# Periodontal Conditions and Pathogens Associated with Pre-Eclampsia: A Scoping Review

**DOI:** 10.3390/ijerph18137194

**Published:** 2021-07-05

**Authors:** Jocelyne Gare, Aida Kanoute, Nicolas Meda, Stephane Viennot, Denis Bourgeois, Florence Carrouel

**Affiliations:** 1 Laboratory P2S (Health Systemic Process), UR4129, University Claude Bernard of Lyon 1, University of Lyon, Lyon, France; jvgare@yahoo.fr (J.G.); stephane.viennot@univ-lyon1.fr (S.V.); denis.bourgeois@univ-lyon1.fr (D.B.); 2 Public Health Laboratory (LASAP), ED2S Doctoral School of Sciences and Health, University Joseph Ki Zerbo, Ouagadougou 7021, Burkina Faso; nicolas.meda@gmail.com; 3 Public Health Service, Department of Dentistry, Faculty of Medicine, Pharmacy and Dentistry, University Cheikh Anta Diop, Dakar 10700, Senegal; aida.kanoute@gmail.com

**Keywords:** periodontal disease, oral microbiota, periodontal pathogens, pre-eclampsia, gingivitis, pregnancy, dysbiosis, placenta, inflammation

## Abstract

Background: Pre-eclampsia, the second most frequent direct source of maternal mortality, is a multisystem gestational disorder characterized by proteinuria and maternal hypertension after the 20th gestational week. Although the causes of pre-eclampsia are still discussed, research has suggested that the placenta has a central place in the pathogenesis of this disease. Moreover, current surveys indicated that periodontal disorders observed during the pregnancy and more particularly, periodontal pathogens could be link to the risk of pre-eclampsia. Objectives: This article aims to review recent studies focusing on periodontal conditions and pathogens associated with pre-eclampsia. Methods: The process followed the Preferred Reporting Items for Systematic Reviews and Meta-Analyses extension for Scoping Reviews guidelines. Results: Metabolic conditions, immunological changes, fluctuating progesterone and estrogen levels of the pregnant woman induce a dysbiosis of the oral microbiota and contribute to increase inflammation of periodontal tissues. Periodontal pathogens could diffuse through the bloodstream inducing a placenta inflammatory response as well as inflammatory molecules produced in response to periodontopathogens could migrate through the bloodstream leading to a placenta inflammatory response. Also, periodontopathogens can colonize the vaginal microbiota through the gastrointestinal tract or during oro-genital contacts. Conclusion: A cumulative bi-directional relationship between periodontal conditions, pathogens and the pre-eclampsia exists.

## 1. Introduction

Pregnancy causes significant unique changes in maternal immune responses and metabolism, yet it is unclear whether/how these alterations may be connected to infections [1]. There is however, strong and solid scientific evidence that pregnancy can induce bacterial dysbiosis, especially in the vaginal and gut microbiome, leading to metabolic alterations and complications in the mother and the newborn [1,2,3]. Hypertension is the most frequent health complication in pregnancy, affecting 10% of pregnancies worldwide [4]. Classification of hypertensive disorders during pregnancy are classified into 4 categories including pre-eclampsia superimposed on chronic hypertension and pre-eclampsia-eclampsia [5]. 

Pre-eclampsia is the second most frequent direct source of maternal mortality, causing an average of 500,000 fetal and neonatal deaths and an estimated of 76,000 direct maternal deaths and each year [6]. Pre-eclampsia complicates 2–8% of pregnancies [5]. Pre-eclampsia, ranged from mild to severe, is a multisystem gestational disorder characterized by proteinuria and maternal hypertension after the 20th gestational week [7]. Although the causes of pre-eclampsia are still discussed, research has suggested that the placenta has a central place in the pathogenesis of this disease. Evidence supports that fetuses and neonates of preeclamptic women are especially impacted by the maternal condition, independently of uteroplacental restriction of flow [8]. Pre-eclampsia is considered to be an endothelial disturbance where the oxidative pathophysiological stress and disturbance of lipid status have potential implication in the development of pre-eclampsia among high-risk pregnancies [9].

There is as scarce new evidence that pregnancy induces dysbiosis in the maternal microbiome in oral cavity [10]. In the 2017, the role of periodontal diseases (PD) on adverse pregnancy outcomes was discussed [11]. PD, classified as a non-communicable disease, is a chronic multifactorial and inflammatory immunological disease of polymicrobial origin resulting from an increase in the pathobionts in the microbiota [12,13]. It should be considered that higher periodontal disease prevalence is found among pregnant women with pre-eclampsia. The "Keystone-Pathogen Hypothesis", of which *Porphyromonas gingivalis* (*P. gingivalis)* plays a key role, postulates that specific bacteria in limited quantities can influence the host immune system and switch the microbiota from symbiotic to dysbiotic to induce inflammatory disorder [14]. The virulence characteristics assigned to these specific periodontal pathogens make them potential contributors in adverse pregnancy outcomes [13,15]. As well, increased proportion of bleeding periodontal sites would induce hematological dissemination of periodontal pathogens and their products, and subsequently would later lead to an immune/inflammatory reaction in the feto-placental unit [16].

A better understanding of the risk factors and processes that cause preeclampsia would allow the identification of women at risk for preeclampsia before the onset of clinical signs. This article focuses on recent studies on periodontal conditions and pathogens associated with pre-eclampsia.

## 2. Materials and Methods

In order to provide an overview of the available research data, a literature review was carried out based on the guidelines of a scoping review [17,18], fulfilling PRISMA-ScR criteria (Preferred Reporting Items for Systematic reviews and Meta-analyses)(Table S1) [19]. The scoping review [20] allows the inclusion of all study designs using the following steps: (1) identification of a clear research objective and search strategies, (2) selection of relevant publications, (3) categorization of the publications, (4) extraction of data, and (5) summarizing, analyzing and reporting the results. 

### 2.1. Identification of Research Question

Research questions were defined to answer the research objectives: “Is the pregnancy associated with periodontal diseases?”, “Are the hormonal oral, immunological and oral microbiota changes and the periodontal disease associated during the pregnancy” and “Are the periodontal disease and the periodontal pathogens associated with the risk of pre-eclampsia?”. The PICO question was: Are pregnant women (P) who have a dysbiotic subgingival microbiota (I) compared with those with a symbiotic subgingival microbiota (C) at increased risk for pre-eclampsia (O)?

### 2.2. Selection of Publications

Electronic research was organized in Pubmed, Embase and Web of science. The following search terms were used: (“pregnant” OR “pregnancy” OR “gravid” OR “expectant”) AND (“hormone” OR “estrogen” OR “progesterone” OR “immunity” OR “microbiota” OR “periodontal” OR “pathogen”) AND (“pre-eclampsia” OR “pregnancy disorder”). Two reviewers (J.G. and A.K.) performed this research. The duplicates were removed.

#### Screening and Eligibility of Publications

Titles and abstracts were reviewed for eligibility. The inclusion criteria were: (i) publications written in English language; (ii) publications published between 2010 and April 2021; (iii) publications presenting human studies; (iv) publications focusing on pregnant woman; (v) publications focusing on periodontal disease or periodontal microbiota; (vii) publications focusing on pre-eclampsia. The exclusion criteria were: (i) pregnant woman with systemic diseases and (ii) congress abstracts or commentaries.

The selection of studies was done independently by the two reviewers based on a screening of titles and abstracts qualified in the electronic database. The selected papers were cross-checked, and any discrepancies were resolved by including a third author (F.C.) to reach a consensus about study inclusion.

### 2.3. Determination of the Association between Periodontal Disease, Periodontal Pathogens and Pre-Eclampsia

#### 2.3.1. Classification of Publications according to the Level of Evidence

From the included publications, the studies were classified by level of evidence [21]: non-experimental studies (i.e., observational studies: case reports, case control studies and cohort studies), experimental studies (i.e., randomized controlled trials) and reviews (i.e., systemic reviews and meta-analyzes).

#### 2.3.2. Data Extraction from the Included Studies, GRADE Classification of Publications and Summary of Results

For each publication in each level of evidence and category, the size of the study population, objectives, study design, results and conclusions were analyzed and coded using the GRADE process. The GRADE process was applied to evaluate the quality of the studies [22,23]. The two reviewers graded independently the publications by using the following levels:

- High: The real effect is similar to that of the estimated effect;
- Moderate: The real effect is likely to be similar to the estimated effect, but it may be considerably different;
- Low: The real effect may be considerably different from the estimated effect.;
- Very low: The real effect is likely to be considerably different from the estimated effect.

In case of disagreement on a GRADE, the 2 reviewers discussed until a consensus was reached.

## 3. Results

### 3.1. Selection of Publications Included 

**Figure 1:**
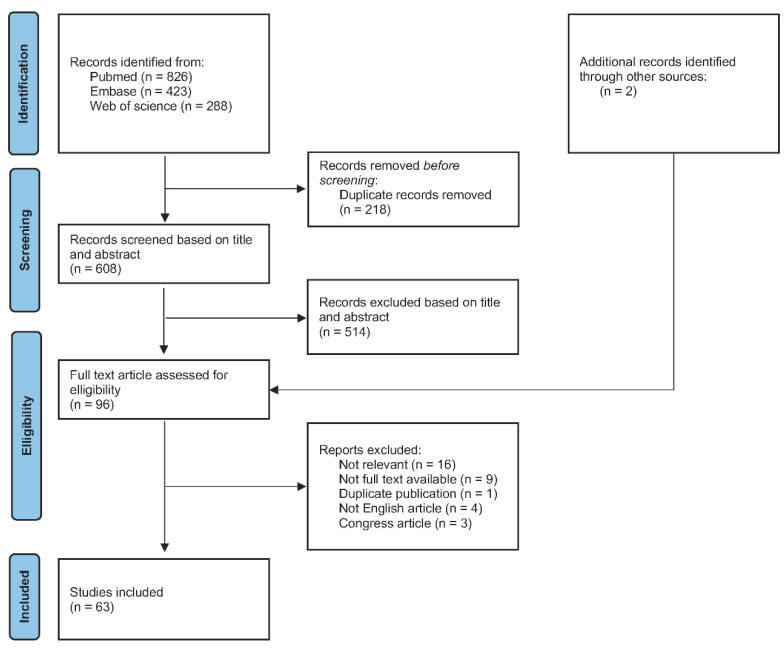
PRISMA-ScR flowchart of study selection process.

From initial database searches, 1537 papers were identified. After removing the duplicates, 608 were screened at the title and abstract levels and 96 papers were retained for full-text assessments. Finally, 72 papers met the inclusion criteria for this review. The PRISMA-ScR study flowchart describing these different steps are presented in Figure 1.

### 3.2. Included Publications Characteristics

Among the 63 studies included, 11 focused on the periodontal conditions during pregnancy, 25 focused on hormonal oral, immunological and oral microbiota changes and their impact on PD during the pregnancy, and 27 focused on periodontal pathogens and pre-eclampsia. The main characteristics of these studies are presented in the Supplementary material Table S2.

### 3.3. Synthesis of the Results

**Figure 2:**
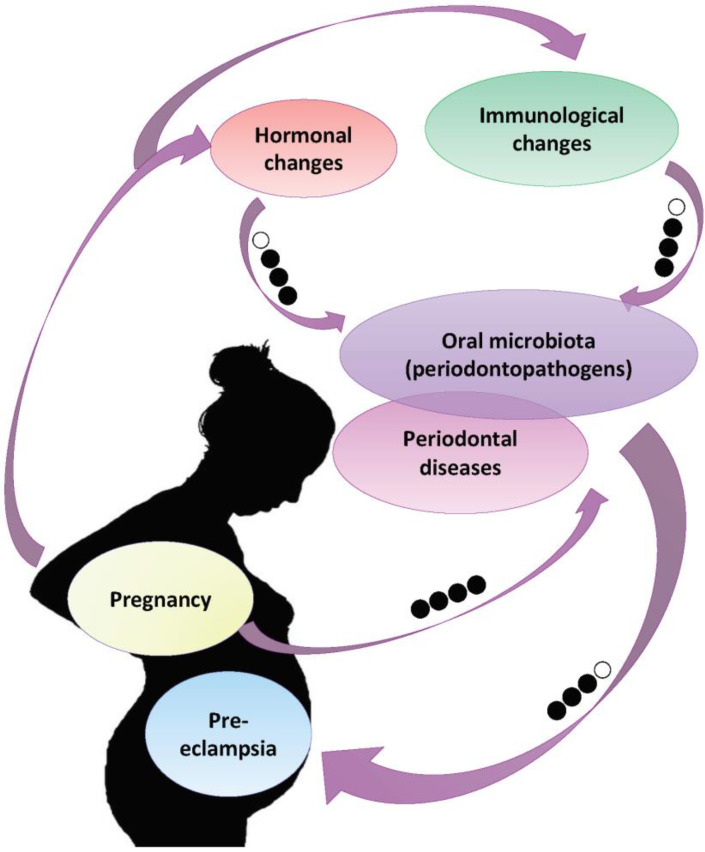
Summary of the results based on the Grade process. High (⬤⬤⬤⬤) when the true effect lies close to that of the estimate of the effect, moderate (⬤⬤⬤◯) when is likely to be close to the estimate of the effect, but there is a possibility that it is substantially different, low (⬤⬤◯◯) when may be substantially different from the estimate of the effect, and very low (⬤◯◯◯) when is likely to be substantially different from the estimate of effect.

The results of the analysis are summarized in Figure 2. A high association between pregnancy and periodontal diseases was observed. Moderate grade was obtained for the association between the hormonal oral, immunological and oral microbiota changes and the periodontal disease. For the association between the periodontal disease, the periodontal pathogens associated with the risk of pre-eclampsia, the determined grade was moderate. 

## 4. Discussion

From this scoping review, preferred to systematic reviews which must be exhaustive when the information on a subject is complex and diverse [20], original information may be highlighted.

### 4.1. Periodontal Conditions during the Pregnancy

Pregnancy gingivitis is defined as a form of PD due to the hormonal changes. It is a common inflammatory pathology of the superficial periodontium that occurs during pregnancy. Inflammation is induced by biofilm and exacerbated by the increased levels of sex steroid hormones characteristic of pregnancy [24]. Clinical symptoms of this mucosal involvement can be generally described as gingivitis and stomatitis of pregnancy [25]. Symptoms usually occur in the second or third month of pregnancy. The gums appear swollen, sensitive, red, increase in volume and bleed easily [26]. As a result, pregnancy gingivitis has been reported to be the most common oral manifestation during pregnancy with a prevalence of 35–100%, depending on the study cohort [27].

Hormonal fluctuations increase blood circulation to the gum tissue with secondary inflammation of the gingival tissue as a result of the presence of dental plaque [28]. In a lower number of cases, gingivitis can be complicated by the development of an epulis [25]. Current studies have indicated that the increase in estrogen and progesterone concentrations in the blood of pregnant women is responsible for the increase of gingivitis [29]. Thus, gingivitis can be induced by many substances and hormones secreted during pregnancy – such as growth hormone, estrogen, vasoactive intestinal polypeptide, progesterone; this result in modifications of the oral mucosa and especially of the gingival tissue [28]. High progesterone levels affect capillaries vessels by inducing proliferation of the endothelium [30].

Risk factors are chronic gingivitis, inadequate oral hygiene, antihypertensive, use of hormonal therapies, antiepileptic, immunosuppressive drugs in pregnancy [31]. Clinicians must be conscious of higher gingival inflammation measures may be found in women who have a higher body mass index [32]. Inflammatory mediators are secreted by adipose tissues that lead to a generalized inflammatory status in the organism of obese pregnancy women. Therefore, these patients may have a significant inflammatory response in the periodontal tissues, and this even in the context of a normal dental plaque [33].

Pregnancy gingivitis should not be neglected because, in the absence of treatment, it can progress to periodontitis, a multifactorial chronic infectious disease inducing an immune-inflammatory response that can lead to tooth mobility and ultimately to the loss of the dental organ itself [34].

PD is classically synonymous with the presence of periodontal pockets corresponding to the distance between the height of the free gingival margin and the epithelial attachment [35]. There is consensus to confirm the increase in depth of probing with progression of the pregnancy. The proportion of sites with a pocket depth greater than 3 mm at 6–8 weeks of pregnancy is significantly higher than at first trimester [36]. However, it must be considered that the increase in periodontal pockets is associated to an increase in the volume of the marginal gingiva rather than to a loss of attachment. While significantly higher levels of probing depth and gingival inflammation were reported during pregnancy, a progressive increase in gingivitis was observed from first to third trimester [24,37]. In most cases, however, these alterations are reversible at the end of pregnancy or 45 days after delivery [28].

### 4.2. Hormonal Oral, Immunological and Oral Microbiota Changes and Their Impact on Periodontal Disease during the Pregnancy

During the gestation, the female body undergoes a series of hormonal, metabolic, and immunological changes [38,39], which may have a significant effect on the composition of the oral microbiome.

During pregnancy, the level of hormones changes [40]. Particularly, the increase of estrogen and progesterone can increase her susceptibility to bacterial plaque provoking the apparition gingivitis that is most frequent during the second to third trimester of pregnancy [41]. The analysis of salivary estrogen levels showed that the estradiol level was ten times higher in pregnant women at the first trimester than non-pregnant women [42]. Salivary estrogen levels increased significantly during the second and third trimesters. In both participant groups, the bleeding on probing was correlated significantly with plaque index, but not with estrogen levels. In all trimesters and postpartum, subjects with high estrogen and PI levels had a higher frequency of gingivitis in pregnancy. During the second and third trimesters, the simultaneous increase in estrogen and PI levels increase the risk of developing gingivitis compared to PI alone. Therefore, during pregnancy, estrogen level determines the magnitude of gingival inflammation developed against microbial plaque at the gingival margin [42].

Progesterone, as its name suggests, is the pregnancy-promoting hormone. Progesterone levels throughout pregnancy increase progressively, reaching concentrations that are ten times higher than those found during the luteal phase of the genital cycle [43]. Gürsoy et al. demonstrated that salivary progesterone concentration increases significantly throughout pregnancy and decreases postpartum. Pregnant women have approximately 18 times higher progesterone level than non-pregnant women [42].

Estrogens and progesterone perform their functions by binding to specific intracellular receptors involved in the regulation of cell growth, differentiation and development [44,45]. Because estrogen receptor and progesterone receptor localization has been reported in the human periodontium, the increase in circulating levels of estrogen and progesterone should have a dramatic effect on the periodontium throughout pregnancy and correlates with the clinical phenomenon [43].

The serum estradiol and progesterone levels increased greatly during pregnancy and were much higher in the pregnant women that in the nonpregnant group [46]. A positive association was found between increased gingival inflammation and increased serum estradiol and progesterone levels during pregnancy [46]. A positive association was also observed between the presence of periodontopathogen such as *P. gingivalis* and the progesterone levels in the first trimester [41]. The hormonal modifications promote the growth of several Gram-negative anaerobic bacteria in the oral cavity such as *Prevotella intermedia* (*P. intermedia*), *Prevotella nigrescens* and *Campylobacter rectus* (*C. rectus*) [10].

The periodontium is a target tissue for estrogens and progesterone that cause vascular, cellular and microbiological changes [47,48]. The elevated levels of estrogens and progesterone act on the gingival vasculature and could cause an increase of erythema, edema, crevicular fluid, and bleeding [41]. Moreover, progesterone increases the synthesis of prostaglandins, particularly prostaglandin E2. Prostaglandins increase vascular capillarity and permeability, thus amplifying the clinical manifestations of gingival inflammation, the gingiva exudates and this exudate allows bacteria to multiply [48]. Progesterone also retards the synthesis of glycosaminoglycans by gingival fibroblasts and thus acts on the inflammatory reaction [49]. On the other hand, estrogens reduce the keratinization of the gingival epithelium and alter the fundamental substance of the connective tissue [50]. The decrease in epithelial keratinization associated with the increase in epithelial glycogen leads to a decrease in the effectiveness of the epithelial barrier [51].

During pregnancy, the immune system is modified to be able to tolerate the fetus. This modification affects the defensive system of periodontal tissues [47]. The increase of sex hormones acts on the function and activity of polymorphonuclear. Impaired neutrophil functions are associated to an increased susceptibility to inflammation [28]. The environment is in an anti-inflammatory state controlled by anti-inflammatory cytokines such as interleukin- (IL) 4, IL-5, IL-10, IL-13, and granulocyte-macrophage stimulating factor [52]. This anti-inflammatory state can be dysregulated and associated to a shift toward the pro-inflammatory cytokines represented by IL-1, IL-2, IL-6, IL-12, IL-15, IL-18, interferon-γ, and tumor-necrosis factor-α [53,54].

**Figure 3:**
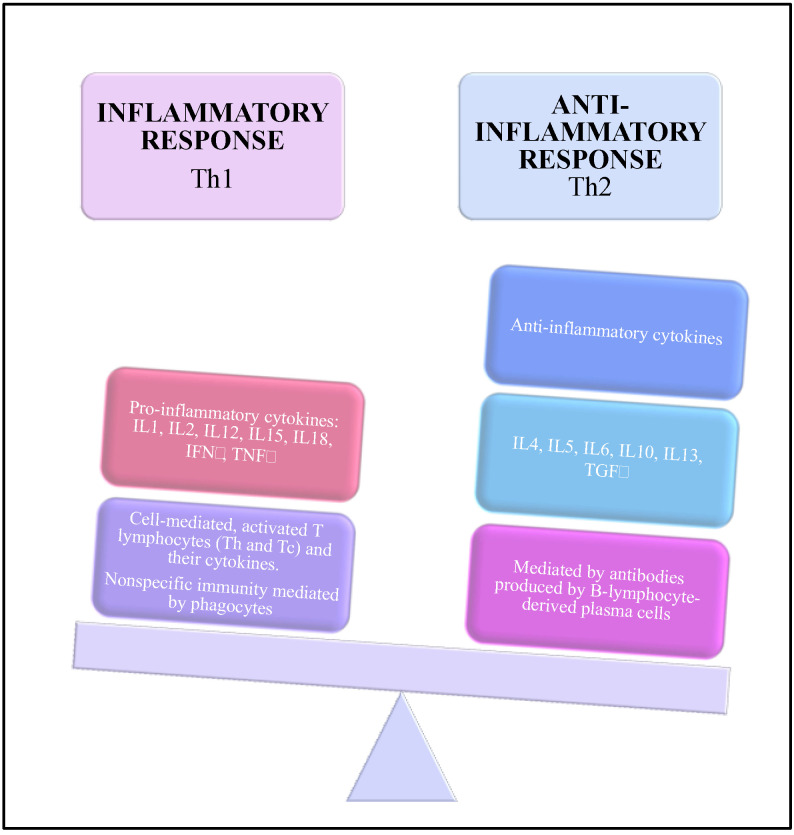
Immunological changes during the pregnancy.

At the systemic level, the immune response of the pregnant woman is associated with an alteration of the balance between cellular immunity (Th1 cytokines) and humoral immunity (Th2 cytokines). Humoral immunity increases while cellular immunity decreases [47] (Figure 3).

Receptors for estrogen have been found in thymocytes and thymic epithelial cells. Estrogen injection is followed by atrophy of the thymus and, therefore, the number of TCD4 and TCD8 lymphocytes is reduced [55]. Also, intracellular and membrane estrogen receptors have been reported in these lymphocytes, so that estrogens reduce the number of TCD4+ and TCD8+ lymphocytes and increase the activity of B lymphocytes, as well as the production of immunoglobulin M and G [56].

Several studies have analyzed the association between the quantity of bacteria (PI) and the gingivitis. In a meta-analysis, Figuero et al. selected 7 studies for analysis of PI [24]. In cohort studies, PI was not significantly modified during pregnancy, but in cross-sectional studies, PI was significantly slightly higher in pregnant women. No differences were found when comparing pregnant and postpartum women. These results are confirmed by the study of Wu et al. [46]. They used a methodology very similar to that of Gürsoy et al. [57], except that participants had excellent plaque control, obtained through oral hygiene instructions throughout the duration of the study. They reported that PI was not significantly modified, as gingival index and bleeding increased significantly in the second and third trimester of pregnancy.

**Figure 4:**
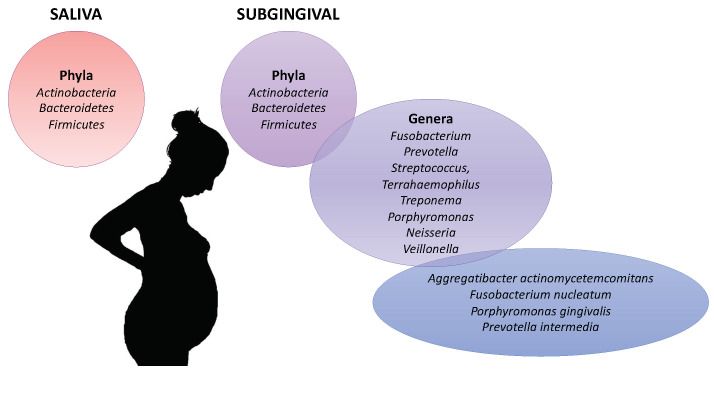
Oral microbiota associated with the pregnancy.

The oral microbiota was compared between pregnant and non-pregnant women (Figure 4) [58,59,60]. Fujiwara et al. analyzed by polymerase chain reaction (PCR) the subgingival microbiota of 132 Japanese pregnant women and 51 Japanese nonpregnant women [60]. The total number of microorganisms was significantly higher in the saliva of pregnant women compared to Japanese non-pregnant [60]. Their microbiota contained higher amounts of *P. gingivalis*, Aa in early and mid-pregnancy. *P. intermedia* and *F. nucleatum* did not change depending on whether women were pregnant or not. *Candida* species were more prevalent in mid and late gestation [60]. Borgo et al. [58] analyzed by quantitative PCR the presence of *A. actinomycetemcomitans*, *P. intermedia*, *P. gingivalis* and *F. nucleatum* in 23 pregnant or 9 non-pregnant women. They showed that *Aggregatibacter actinomycetemcomitans* (*A. actinomycetemcomitans*) was detected in significant higher amounts in the second trimester and in the third trimester of gestation [58]. *F. nucleatum* and *P. intermedia* was detected in high level in pregnant women whereas *P. gingivalis* was detected in both pregnant and non-pregnant [58]. Lin et al. observed in pregnant women, a higher abundance of *Treponema*, *Porphyromonas* and *Neisseria*, while in the non-pregnant women, *Streptococcus* and *Veillonella* were more abundant [61]. Balan et al. also observed that the oral microbiota was composed of a higher abundance of pathogenic species (*Prevotella*, *P. gingivalis* and *F. nucleatum*) in healthy pregnant as compared with nonpregnant one whereas they had similar gingival and plaque index scores [62].

The evolution of the oral microbiota during the gestation was studied. In pregnant women, DiGiulio et al. observed that the average taxonomic composition of the saliva remained constant over gestational time [63]. Balan et al. also observed that subgingival and saliva microbiota were relatively stable in terms of species richness and diversity during the course of pregnancy [62]. However, they observed the increasing of pathogenic bacterial during pregnancy. In pregnant women, members of phyla *Actinobacteria*, *Bacteroidetes* and *Firmicutes* dominated in both saliva and subgingival samples. The genera *Fusobacterium*, *Prevotella*, *Streptococcus*, *Terrahaemophilus* and *Veillonella* were most abundant in subgingival microbiota. Concordant results were observed in pregnant Chinese women [61]. During the pregnancy, a higher abundance of *Prevotella* species, *P. gingivalis*, and *F. nucleatum* was observed [62]. 

The composition of the oral microbiota was also compared between pregnant women suffering or not of gingivitis. Yang et al., in a pilot study, concluded that the gingivitis in pregnant women was not correlated with a shift in the overall composition or diversity of the subgingival microbiota [54]. The main phyla observed in the subgingival microbiota of both groups were *Actinobacteria*, *Bacteroidetes*, *Firmicutes*, *Fusobacteria*, *Proteobacteria*, and *Spirochaetes*. However, they observed modification of several bacteria taxa in pregnant women suffering o gingivitis compared to healthy pregnant women. Particularly, 5 operational taxonomic units contained species known as periodontal or opportunistic pathogens. These unbalance between commensal bacteria and pathogenic bacteria is associated, the dysbiosis of the subgingival microbiota and with the apparition of gingivitis [64]. In 2014, Tellapragada et al. analyzed by PCR the subgingival plaque of pregnant women and demonstrated an association between gingivitis and the presence of *C. rectus*, *P. gingivalis*, *P. intermedia*, *Parvimona nigrescens* and *Treponema denticola* (*T. denticola*) [65]. 

After the delivery, the abundance of pathogenic species decreased was accompanied by simultaneous repopulation of healthy microbiome such as *Lautropis mirabilis* sp., *Rothia aeria*, *Granulicatella adiacens* and *SR1* sp [62]. These species have been reported to be dominating in the health-associated microbial communities in healthy subjects in previous studies [66]. Pregnancy-related transition to pathogenic microbiome and its restoration to health during the postpartum period could be a result of complex host–microbial interactions that may be taking place under the influence of hormonal and immunological factors [28]. In addition, pregnancy-induced perturbations in the oral cavity may disrupt the ecological balance maintained by interspecies interactions [10]. These may trigger the overgrowth of species with pathogenic potential and suppress the healthy microbiome [10]. 

### 4.3. Periodontal Pathogens and Pre-Eclampsia

In pregnant women, the PD and more particularly, the presence of periodontal pathogens has been associated to adverse pregnancy outcomes such as pre-eclampsia [10,64,67,68]. Several meta-analyses concluded that maternal PD was an independent predictor of pre-eclampsia [69,70,71,72,73,74]. Three pathways could explain this association [12,64]. Firstly, periodontopathogen that are mobile bacteria could migrate, invade the epithelium, the connective tissue, reach the bloodstream and diffuse into the body. Thus, adhesion proteins expressed on the surface of the bacteria can bind to the placental cell receptors and trigger a downstream inflammatory response [75]. Secondly, inflammatory molecules produced in response to periodontopathogens could migrate through the blood stream. Finally, periodontopathogens can reach and colonize the vaginal microbiota through the gastrointestinal tract or during oro-genital contacts [10,64].

Pre-eclampsia is a placental dysfunction due to early angiogenic and inflammatory dysregulation. Clinically, it is characterized by the new appearance of hypertension, end organ dysfunction and potential proteinuria after 20 weeks of gestation. The consequences can be the maternal morbidity or adverse fetal outcomes such as intra-uterine growth restriction, preterm birth, placental abruption, fetal distress, and fetal death in utero [76]. Even if the real cause of pre-eclampsia is not elucidated, it appears that dysbiosis of the placental microbiota could be a key risk factor [77,78].

While in normotensive women, no bacteria could be detected by PCR in placental samples, 12.7% of samples from women with pre-eclampsia had bacteria. The bacteria identified included *Bacillus cereus*, *Escherichia*, *Listeria*, *Salmonella* (usually associated with gastrointestinal infection); *Anoxy bacillus* and *Klebsiella pneumonia* (usually associated with respiratory tract infections); and *Dialister*, *Porphyromonas*, *Prevotella*, and *Variovorax* (usually associated with periodontitis) [79]. The comparative metagenomic analysis on 320 placental specimens revealed that the placental microbiome was closely related to the supragingival plaque [78]. Concordant results were obtained by Barak et al. [80]. 50% of placenta samples from pregnant women suffering of pre-eclampsia had periodontopathogens such as *Actinobacillus actinomycestemcomitans*, *F. nucleatum*, *P. gingivalis*, *P. intermedia*, *Tannerella forsynthia*, and *T. denticola* (*Spirochaetes*) in the placentas of women with pre-eclampsia. 

The presence of periodonthopathogens that induce PD conduce to the increase of pro-inflammatory cytokines and thus the dysregulation of the normal immunological state during pregnancy [81]. Indeed, pregnant women suffering of PD presented a high level of plasma C-reactive protein that is the witness of systemic inflammation [54]. An increase of the level of pro-inflammatory cytokines in the peripheral circulation was also observed in case of PD [82]. In pre-eclamptic women with periodontitis, the presence of *P. gingivalis* and *P. intermedia* was associated with the increase of TLR-4 and NF-κB expression in the placenta [83]. 

In addition, during placental development, to ensure androgen signaling and epigenetic regulation of gene expression, the placenta utilizes histone lysine demethylases and androgen receptors [84]. Thus, disruption of androgens could be the cause of abnormal placental development. And since a study showed that *P. intermedia* and *P. gingivalis* can reduce testosterone to 5-alpha dihydrotestosterone (DHT) and induce DHT synthesis by fibroblasts [85] then these bacteria could interfere with androgen signaling and deregulate placental development.

Periodontopathogen could also be pathogens of the placenta bed. The favorable outcome of pregnancy is associated with uterine vascular changes with, in particular, the adequate remodeling of the uterine spiral arteries. Inappropriate remodeling of the myometrial segments of the uterine spiral arteries is termed defective deep placentation [86,87]. The defective deep placentation has been demonstrated in case of pre-eclampsia [87]. During the first trimester of pregnancy, extravillous trophoblasts form anchoring villi in order to attach the placenta to the decidua and the uterine wall which will allow the transmission of pathogens from mother to fetus [88]. The presence of *P. gingivalis* was detected in 70–92% of samples from the inner third of the placental bed - known as the decidua - of women with pre-eclampsia [89,90]. *P. gingivalis* was detected within the villous stroma or umbilical cord. Its presence in the umbilical cord was significantly associated with pre-eclampsia [91]. Several in vitro and in vivo studies realized in rat or primate’ models indicate that *P. gingivalis* can impact the remodeling of the uterine spiral arteries [92,93,94].

### 4.4. Oral Lifestyles as an Actor to Reduce the Incidence of Periodontal Pathogens and Periodontal Disease

Since periodontal bacteria and PD represent a risk of preeclampsia for the pregnant woman, it is therefore essential to implement a maintenance plan for women gradually to reduce the incidence of periodontal lesions and the resulting damage.

To fight against the dysbiosis of oral microbiota through the mechanical disruption of the bacterial plaque, the solution is the oral hygiene and the preventive dentistry [95]. To remove the subgingival microbiota, a professional oral hygiene therapy is required. However, the daily individual oral hygiene is indispensable to prevent the accumulation of microbiota and its dysbiosis [96,97]. In order to achieve this home management of bacterial plaque, the toothbrushes appear as indispensable tools. Toothbrushes can be manual but the mastery of brushing techniques is essential to have an optimal action against dental plaque. On the other hand, electric toothbrushes allow you to eliminate plaque without mastering the technique [98,99]. Moreover, the use of electric and sonic toothbrushes has demonstrated their efficacity to reduce the incidence of inflammation [100]. Within the framework of individual prophylaxis to disorganize the biofilm, it is necessary, in addition to a good brushing technique, to emphasize the importance of managing the interdental space. Toothbrushing alone is insufficient because it does not allow to act on the interdental biofilm which even in young clinically healthy subjects contains periodontopathogenic and cariogenic bacteria [101,102]. Interdental cleaning aids such as dental floss, interdental brushes (IDB) or toothpicks should be included in the oral hygiene routine to control the interdental microbiota and prevent dysbiosis. Clinical studies have highlighted the effectiveness of flossing [103,104]. The use of IDBs has also shown to be effective in restoring interdental microbiota symbiosis [105] and controlling gingival inflammation [106]. IDBs have been shown to be more effective than flossing in managing gingivitis [107,108].

The control of the oral microbiota can also be managed through a chemical action by using toothpastes or mouthwashes. Indeed, they contain molecules that can have antibacterial and anti-inflammatory activities [109]. The oral care cosmetics containing silver, gold, zinc oxide, titanium dioxide, copper oxide … act against the plaque accumulation and the gingivitis [109]. Herbal oral care products were also effective in the control of plaque and gingivitis [110]. 

The rigorous control of supragingival or even subgingival bacterial plaque is the central element of periodontal therapy and the best means of prevention to date. Mouthwashes based on Chlorhexidine have been developed for their antibacterial action [109]. However, their effectiveness in reducing plaque, inflammation and gingival bleeding is still debated, especially after scaling and root planning interventions, such as antimicrobial strategies. As an alternative to the use of Chlorhexidine in mouthwash, which has adverse effects, probiotics represent a significant advance in the prevention and care of periodontitis [111]. Also, some probiotics are thought to have a function in the maintenance of periodontal health and the treatment of PD [112]. Probiotic medicine is based on the concept of a normal and healthy microflora [113]. Oral administration of *Lactobacillus reuteri*, *Lactobacillus salivarius* and *Lactobacillus brevis* had a positive impact on the clinical signs of PD and reduced the levels of major periodontal pathogens [114]. However, the benefits were maintained only with continuous probiotic administration. In this sense, new probiotic formulations recently marketed in toothpaste and chewing gum are of considerable interest. Recently commercialized, the combination of *Lactobacillus and Bifidobacterium* in an experimental toothpaste tested could have a synergistic effect [111].

## 5. Conclusions

Although the main limitation of this study is the diversity of the populations included (age, duration of gestation, number of pregnancies...), this review allows to conclude that fluctuating progesterone and estrogen levels and the modification of the immune response during pregnancy impact the subgingival microbiota and contribute to increase the risk of apparition of PD. Moreover, the PD and more particularly, the periodontal pathogens increase the risk of pre-eclampsia.

## Supplementary Materials

The following are available online at https://www.mdpi.com/article/10.3390/ijerph18137194/s1, Table S1: Preferred Reporting Items for Systematic reviews and Meta-Analyses extension for Scoping Reviews (PRISMA-ScR) Checklist, Table S2: Characteristics of included studies.
