# Supplementary material for: Periodontal Conditions and Pathogens Associated with Pre-Eclampsia: A Scoping Review
Source: Int J Environ Res Public Health. 2021 Jul 5;18(13):7194. doi: 10.3390/ijerph18137194 (PMC8297070; doi:10.3390/ijerph18137194)
Supplement: Supplementary file 1 [file ijerph-18-07194-s001.zip › ijerph-1269461-supplementary.pdf]

Table S1. Preferred Reporting Items for Systematic reviews and Meta-Analyses extension for Scoping Reviews (PRISMA-ScR) Checklist

SECTION	ITEM	PRISMA-ScR CHECKLIST ITEM	REPORTED ON PAGE #
**TITLE**			
Title	1	Identify the report as a scoping review.	1
**ABSTRACT**			
Structured summary	2	Provide a structured summary that includes (as applicable): background, objectives, eligibility criteria, sources of evidence, charting methods, results, and conclusions that relate to the review questions and objectives.	1
**INTRODUCTION**			
Rationale	3	Describe the rationale for the review in the context of what is already known. Explain why the review questions/objectives lend themselves to a scoping review approach.	1-2
Objectives	4	Provide an explicit statement of the questions and objectives being addressed with reference to their key elements (e.g., population or participants, concepts, and context) or other relevant key elements used to conceptualize the review questions and/or objectives.	2
**METHODS**			
Protocol and registration	5	Indicate whether a review protocol exists; state if and where it can be accessed (e.g., a Web address); and if available, provide registration information, including the registration number.	3-4
Eligibility criteria	6	Specify characteristics of the sources of evidence used as eligibility criteria (e.g., years considered, language, and publication status), and provide a rationale.	3
Information sources*	7	Describe all information sources in the search (e.g., databases with dates of coverage and contact with authors to identify additional sources), as well as the date the most recent search was executed.	3
Search	8	Present the full electronic search strategy for at least 1 database, including any limits used, such that it could be repeated.	3
Selection of sources of evidence†	9	State the process for selecting sources of evidence (i.e., screening and eligibility) included in the scoping review.	3
Data charting process‡	10	Describe the methods of charting data from the included sources of evidence (e.g., calibrated forms or forms that have been tested by the team before their use, and whether data charting was done independently or in duplicate) and any processes for obtaining and confirming data from investigators.	3

SECTION	ITEM	PRISMA-ScR CHECKLIST ITEM	REPORTED ON PAGE #
Data items	11	List and define all variables for which data were sought and any assumptions and simplifications made.	3
Critical appraisal of individual sources of evidence§	12	If done, provide a rationale for conducting a critical appraisal of included sources of evidence; describe the methods used and how this information was used in any data synthesis (if appropriate).	NA
Synthesis of results	13	Describe the methods of handling and summarizing the data that were charted.	3
**RESULTS**			
Selection of sources of evidence	14	Give numbers of sources of evidence screened, assessed for eligibility, and included in the review, with reasons for exclusions at each stage, ideally using a flow diagram.	3-4
Characteristics of sources of evidence	15	For each source of evidence, present characteristics for which data were charted and provide the citations.	Appendix Table S1
Critical appraisal within sources of evidence	16	If done, present data on critical appraisal of included sources of evidence (see item 12).	NA
Results of individual sources of evidence	17	For each included source of evidence, present the relevant data that were charted that relate to the review questions and objectives.	Appendix Table S1
Synthesis of results	18	Summarize and/or present the charting results as they relate to the review questions and objectives.	4-5
**DISCUSSION**			
Summary of evidence	19	Summarize the main results (including an overview of concepts, themes, and types of evidence available), link to the review questions and objectives, and consider the relevance to key groups.	5-11
Limitations	20	Discuss the limitations of the scoping review process.	12
Conclusions	21	Provide a general interpretation of the results with respect to the review questions and objectives, as well as potential implications and/or next steps.	12
**FUNDING**			
Funding	22	Describe sources of funding for the included sources of evidence, as well as sources of funding for the scoping review. Describe the role of the funders of the scoping review.	12

From: Tricco AC, Lillie E, Zarin W, O'Brien KK, Colquhoun H, Levac D, et al. PRISMA Extension for Scoping Reviews (PRISMAScR): Checklist and Explanation. Ann Intern Med. 2018;169:467–473. doi: 10.7326/M18-0850.

Table S2. Characteristics of included studies. BI: bleeding index, CAL: Clinical attachment level, CRP: C-reactive protein, GI: Gingival index, IDB: Interdental brush, MMP: Matrix metalloproteinase, MPO: Myeloperoxidase, MTB: Manual toothbrush, N/A: Not applicable, NP: Non pregnant woman, OR: Odds ratio, P: Pregnant woman, PE: Pre-eclampsia, PD: Periodontal disease, PI: Plaque index, PP: Post-partum woman, PPD: Pocket probing depth, TIMP: Tissue inhibitor of matrix metalloproteinase

Author	Year	Design	Population	Study Aim	Main Findings
Figuero [24]	2013	Systematic review	P, NP, PP	To quantitatively estimate the association between pregnancy and GI	A significantly lower GI in pregnant women in the first term compared with those in their second or third term of pregnancy<br>A significantly lower GI in pregnant women in the first term compared with those in their second or third term of pregnancy<br>GI was significantly lower in P (1st term) compared with P (2nd or 3rd term)<br>GI score was lower in PP women compared with P (2nd term)<br>GI was lower in NP compared with P (2nd or 3rd term)
Cristi [25]	2019	Observational study	N=228, P (different gestational ages)	To evaluate the frequency of epulis gravidarum according to nasal and oral symptoms	Epulis risk was increased in P presenting nasal and gum disorders
Figueiredo [26]	2019	Retrospective cohort study	N=142, P (16th week until the end of gestation)	To investigate the repercussion of PD in the P health and the complications during pregnancy and delivery	PD increased the risk of neonatal and maternal negative outcomes
Onigbinde [27]	2014	Observational study	N=415, P (different gestational ages)	To determine the association of so variables and the periodontal statu in P	The oral hygiene index simplified was significantly increased with the trimester of pregnancy
Wu [28]	2015	Literature review	N/A	To analyze the effects of progesterone and estrogen on the change of subgingival microbiota and immunologic physiological mediators in periodontal tissue	The increased plasma levels of estrogen and progesterone was associated with a decline in periodontal health status, the increasing of the influence of subgingival microbiota and a spectrum of inflammatory responses in gingival tissues
Preethi [29]	2015	Literature review	N/A	To analyze the effect of estrogen and progesterone on oral mucosa and PD	The estrogen and progesterone modified metabolism and immunology of the body and increased the risk of PD
Yunita [31]	2020	Cross-sectional study	N=192, P (different gestational ages)	To determine the association between self-perceived oral health problems and professionally diagnosed oral health status in P	The prevalence of PD among P in this study were relatively high
da Silva [32]	2021	Systematic review (82 cross-sectional/clinical	N/A	To analyze the association between clinical measures of gingival inflammation and body mass index	The higher measures of gingival inflammation are associated with higher body mass index

		trials, 3 case-controls, 5 cohorts)			
Silva de Araujo Figueiredo [34]	2017	Literature review	N/A	To depict the main oral diseases that are related to pregnancy; to clarify some of the possible systemic mechanisms that are associated with these changes; and to address issues about oral care during pregnancy	The most frequent oral manifestations during the pregnancy are: pyogenic granuloma, gingivitis, and periodontitis<br>No change in the amount of oral biofilm was observed during pregnancy<br>The oral biofilm increased the risk and the severity of PD during pregnancy
González-Jaranay [36]	2017	Cohort study	N=96, P (8-10 weeks, 21-23 weeks and 34-36 weeks of gestation) and at 40 days PP	To analyze periodontal status at successive stages of pregnancy and 3-6 weeks PP in women with initial periodontal alterations	PI, GI and Percentage of sites with Probing Depth >3 mm increased during pregnancy and decreased PP
Foratori-Junior [37]	2020	Cross-sectional study	N=100, obesity/ overweight P, normal weight P	To evaluate the association between pre-pregnancy overweight/obesity, periodontitis during the third trimester of pregnancy, and the infants' birth weight	PPD and CAL was higher in obesity/overweight P compared with normal weight P
Lain [38]	2007	Literature review	N/A	To describe metabolic changes during pregnancy	Early gestation was an anabolic state in the mother with an increase in maternal fat stores and small increases in insulin sensitivity<br>Late pregnancy was a catabolic state with decreased insulin sensitivity (increased insulin resistance)
Wang [39]	2016	Cross-sectional study	N=4260, P (n=322), NP	To analyze the effects on systemic metabolism from the alterations in maternal glucose and lipid balance	The multiple metabolic measures (intermediate-density, low-density and high-density lipoprotein triglyceride, fatty acids, amino acids, low-grade inflammatory marker glycoprotein acetyls, IL-18) increased in magnitude across the three trimesters
Machado [40]	2012	Case-control study	N=40, P (n=20, 14-24 weeks of gestation), NP (n=20)	To test the hypothesis of qualitative and quantitative differences of 8 periodontopathogens between pregnant and non-pregnant women	The clinical parameters showed no significant differences between P and NP.<br>The numbers of subgingival periodontopathogens were not found to be significantly different between groups, despite the higher mean counts of *P. intermedia* in P
Massoni [41]	2019	Cohort study	N=67, P (n=16, 1st trimester), P (n=21, 2nd trimester), P (n=15, 3rd trimester), NP (n=15)	To identify quantitatively and qualitatively the subgingival flora in different gestational trimesters, compared to NP To evaluate the correlations between epidemiological characteristics, clinical diagnosis, microbiological findings and	*T. forsythia* was highly prevalent during the 1st trimester and was correlated with the diagnosis of gingivitis among P. *P. gingivalis* was positively correlated with progesterone levels in the 1st trimester.<br>Gestation was positively correlated with the total amount of bacteria, without influence of the hormonal levels or the epidemiological factors evaluated<br>*T. forsythia* favored occurrence of gingivitis during pregnancy

				levels of estradiol and progesterone	The progesterone levels in the 1st trimester enhanced the growth of *P. gingivalis*
Gürsoy [42]	2016	Cohort study	N=30, women followed at three time points during pregnancy and twice PP	To analyze salivary levels of human beta-defensin (hBD)-1, -2, -3, and human neutrophil peptide (HNP)-1 in P, and their relation to periodontal status	The salivary concentrations of hBD-1, hBD-2, and HNP-1 were reduced especially during the 3rd trimester,<br>The hBD-3 concentrations did not change during pregnancy and post-partum visits<br>Weak associations were observed between salivary defensin and hormone concentrations and clinical parameters
Preshaw [43]	2013	Literature review	N/A	To analyze the association between hormone and the periodontum	The increase in circulating levels of estrogen and progesterone should have a dramatic effect on the periodontium throughout pregnancy
Wu [46]	2016	Case-control study	N=50, P (n=30), NP (n=20)	To evaluate the effects of hormonal changes during pregnancy on gingival inflammation and interleukin-1β (IL-1β) and tumor necrosis factor-α (TNF-α) in gingival crevicular fluid (GCF)	PI, BI and GI increased significantly during pregnancy<br>No significant changes in PI, CAL, IL-1β, or TNF-α GCF levels<br>IL-1β, not TNF-α, was higher in P group than in NP group<br>No correlation with serum hormone levels was observed during pregnancy. GI and BI showed significant positive correlation with serum hormone levels during pregnancy
Saadaoui [10]	2021	Literature review	N/A	To summarize the dynamics of oral microbiome during pregnancy and to discuss the relationship between a dysbiotic oral microbiome and pregnancy complications	Sex hormones may cause a significant shift in the oral microbiome composition which leads to dysbiosis and an altered immune response<br>Chronic periodontal infections can cause both local and systemic inflammatory responses
Abu-Raya [47]	2020	Literature review	N/A	To describe the dynamic changes occurring in the peripheral maternal immune system during normal pregnancy	During pregnancy, the immune system is modified to be able to tolerate the fetus<br>This modification affects the defensive system of periodontal tissues
Markou [48]	2009	Literature review	N/A	To analyze the influence of sex steroid hormones on gingiva of women	Some oral micro-organisms synthesize enzymes needed for steroid synthesis and catabolism<br>During pregnancy, the production of sex steroid hormones was increased which resulted in increased gingival inflammation, characterized by gingival enlargement, increased gingival bleeding and crevicular fluid flow and microbial changes
Jafri [50]	2015	Case reports	N=2, P (n=1, 6 months of gestation), NP (n=2)	To analyze how a same hormone at different age and stage shows an exaggerated gingival response to plaque	Female sex hormones are implicated in the changes in periodontal conditions<br>The gingival inflammation is exacerbated during puberty, pregnancy and at the postmenopausal stage<br>A same hormone at different age and stage shows an exaggerated inflammatory response to the plaque<br>Strict oral hygiene maintenance is of high importance
Mascarenhas [51]	2003	Literature review	N/A	To address the link between sex hormones and the periodontium<br>To analyze how these hormones influence the periodontium at different life times	Sexual hormones play a key role in PD progression and wound healing. During pregnancy, there is an increase of PD<br>The influence of sex hormones can be minimized with good plaque control

Mor [52]	2017	Literature review	N/A	To analyze the immunological responses at the receptive maternal–fetal interface<br>To discuss the role of the microbiota in promoting a tolerogenic maternal immune system	During pregnancy, there is an immune suppression and consequently a period of increased risk of bacterial and viral infection<br>Interferon-β is a crucial immune modulator during pregnancy; it protects the fetus against viral infections and contributes to the process of immune regulation at the maternal–fetal interface<br>The immune response associated with placental viral infections can affect maternal and fetal survival
Challis [53].	2009	Literature review	N/A	To analyze inflammation process during pregnancy	During pregnancy, the balance of Th1 and Th2 cytokines is characterized by an initial prevalence of Th2 cytokines, followed by a progressive shift toward Th1 predominance late in gestation<br>Hypoxia and the innate immune response are 2 adaptive mechanisms by which organisms respond to perturbation in organ function, playing a major role in spontaneous PE
Yang [54].	2019	Case-control study	N=34, P (n=34, 1st trimester) with healthy gingiva (n=22) or gingivitis (n=12)	To generate preliminary data about the subgingival microbiome of pregnant, African American women<br>to explore associations among the microbiome, periodontal inflammation and preterm birth	No differences in overall microbiome diversity between the healthy gingiva and the gingivitis groups were observed<br>Significant differences were found among the bacterial taxa present<br>The gingivitis group had higher levels of salivary IL-1β and MMP-8, while CRP was not different between groups<br>Microbiome diversity was positively associated with CRP level
Gürsoy [57]	2014	Cohort study	N=30, P (10±1 weeks of gestation)	To define inflammatory proteins in saliva, induced or inhibited by estradiol, as early diagnostic biomarkers or target proteins in relation to pregnancy-associated gingivitis	In saliva, estradiol concentrations were associated positively with TIMP-1 and negatively with MPO and MMP-8 concentrations
Borgo [58]	2014	Case-control study	N=32, P (n=23, 2nd or 3rd trimester), NP (n=9)	To determine the gingival conditions and the quantitative detection for *A. actinomycetemcomitans, F. nucleatum, P. gingivalis* and *P. intermedia* in pregnant women	A significant relationship with the presence of *A. actinomycetemcomitans* among pregnant women at 2nd and 3rd trimester<br>A significant increase of the gingival inflammation was observed between the 2nd and 3rd trimesters of pregnancy<br>An increase in PI, GI, BI, PPD, and CAL values was observed in P compared with NP<br>During pregnancy, *P. intermedia* were observed in higher levels compared to *P. gingivalis*<br>A high incidence of *F. nucleatum* during the 2nd and 3rd trimester of pregnancy, as well as in NP was observed
Nuriel-Ohayon [59]	2016	Literature review	N/A	To describe the microbial changes that occur in the pregnant female, as well as provide an overview of the initial exposure of the fetus to microbiota—from the placenta, through birth and infancy	The total viable count was higher in P compared with NP<br>During the pregnancy, *P. gingivalis, A. actinomycetemcomitans* and *Candida* were increased<br>The dysbiosis of the microbiota would be associated with adverse pregnancy outcomes

Fujiwara [60]	2017	Case-control study	N=183, P (n=132), NP (n=51)	To examine the changes in the oral microbiota between pregnancy and nonpregnancy periods	The total cultivable microbial counts in the early pregnancy were significantly higher than that of the NP<br>The incidences of *P. gingivalis* and *A. actinomycetemcomitans* in gingival sulcus during the early and middle pregnancy were significantly higher than the NP group<br>*P. intermedia* and *F. nucleatum* did not change during pregnancy<br>*Candida* species were more frequent during the middle and late pregnancy
Lin [61]	2018	Case-control study	N=18, P (n=11, <42 weeks of gestation), NP (n=7)	To explore the bacterial diversity and ecological shifts in the supragingival plaques of P	There was distinct clustering according to gestational status (33 genera may contribute)<br>*Neisseria, Porphyromonas,* and *Treponema* were over-represented in P<br>*Streptococcus* and *Veillonella* were more abundant in NP<br>53 operational taxonomic units were observed to have positive correlations with sex hormones, with *Prevotella* spp. and *Treponema* spp. being most abundant
Balan [62]	2018	Case-control study	N=24, P (n=10 each across the 3 trimesters), PP (n=10)	To analyze the variations in the oral microbial composition and diversity with the progression of pregnancy and in the PP period	The oral microbial diversity was stable during pregnancy and PP<br>The microbiome makes a pathogenic shift during pregnancy and reverts back to a healthy microbiome during the PP<br>During pregnancy, a higher abundance of pathogenic taxa from genera *Prevotella, Streptococcus* and *Veillonella* in both subgingival placa and saliva samples was observed<br>During pregnancy, a higher abundance of *Prevotella* species, *P. gingivalis*, and *F. nucleatum* was observed<br>A significant decrease in the abundance of pathogenic species in P compared with NP was observed<br>Bacterial communities have different co-occurrence and co-exclusion relationships during pregnancy and postpartum period
DiGiulio [63]	2015	Case-control study	N=49, P (n=49 among them, 15 delivered preterm)	To analyze the microbiota compositional dynamics during and after pregnancy	Microbiota community (vagina, distal gut, saliva, and tooth/gum) taxonomic composition and diversity remained remarkably stable
Cobb [64]	2017	Literature review	N/A	To review the oral, vaginal, and placental microbiomes and consider their potential impact on adverse pregnancy outcomes	An association between the dissemination of pathogenic bacteria was associated with moderate and severe periodontitis and extraoral infections and inflammation<br>Specific oral pathogenic bacteria, for example, *F. nucleatum*, *P. gingivalis*, *F. alocis*, *C. rectus*, and others, represented potential collaborators in adverse outcomes of pregnancy<br>The association between PD, the inherent bacteria and systemic impact of inflammatory mediators, and adverse pregnancy outcomes involves several pathways
Tellapragada [65]	2014	Cross-sectional study	N=390, P (n=390, 8–24 weeks of gestation)	To study the periodontal infections among asymptomatic P and to find an association of bacterial etiologies with the disease	Prevalence of gingivitis was 38% and clinical periodontitis was 10% among the study population<br>Among the periodontitis group, high detection rates of *P. gingivalis* (56%), *P. nigrescens* (44%), *T. denticola* (32%), and *P. intermedius* (24%) were noted along with significant association with the disease

Abusleme [66]	2013	Case-control study	N=32, Subjects with PD (n=22), healthy (n=10)	To better understand the ecology of oral subgingival communities in health and periodontitis and elucidate the relationship between inflammation and the subgingival microbiome	In PD, presence of bleeding was not associated with different α-diversity or with a distinct microbiome<br>In PD, bleeding sites showed higher total bacterial load<br>Communities in health and periodontitis largely differed, with higher diversity and biomass in PD<br>Shifts in community structure from health to PD resembled ecological succession, with emergence of newly dominant taxa in PD without replacement of primary health-associated species<br>PD was associated with higher proportions of *Spirochetes, Synergistetes, Firmicutes* and *Chloroflexi*<br>*Actinobacteria*, particularly *Actinomyces*, were higher in health
Wei [67]	2013	Meta-Analysis	N=1089 PE patient	To ascertain the relationship between PD and PE	The causality remains unclear<br>The association between PD and PE may reflect the induction of PD by the pre-eclamptic state, or it may be part of an overall exaggerated inflammatory response to pregnancy
Fischer [68]	2019	Literature review	N/A	To describe the placenta colonization with periodontal pathogens	Periodontal pathogens in placental tissue were associated with multiple adverse pregnancy outcomes<br>Both placental and oral microbiomes may play a role in periodontitis-associated adverse pregnancy outcomes
Konopka [69]	2020	Systematic review	N=2724, P (ampng them 195 suffered of PE)	To review systematic cohort and randomized trials on the relationship between periodontitis and PE	A significant relationship between PD and the risk of PE was demonstrated in 5 cohort trials, which was not confirmed by only 1 study<br>The impact of non-surgical treatment (scaling and root planing) on the occurrence of PE was not confirmed
Daalderop [70]	2018	Systematic review	N/A	To study the association between PD and adverse pregnancy outcomes	Systematic reviews with the lowest risk of bias consistently demonstrated positive associations between PD and PE (odds ratio, 2.2; 95% CI, 1.4 to 3.4; 15 studies, 5,111 participants)<br>Estimated population-attributable fractions for PD were 10% to 55% for PE
Matevosyan [71]	2011	Meta-analysis	N/A	To elucidate plausible associations between PD and pregnancy events	Women with PE have poor periodontal parameters in both, treatment and placebo groups (OR 1.94–2.9)<br>Higher rates of tobacco use (RR 3.02), bacterial vaginosis (RR 2.7), clinical attachment level (OR 2.76), and fetal tyrosine kinase (OR 1.6) contribute in increased rates of PE (RR 1.68)
Sgolastra [72]	2013	Meta-analysis	N/A	To study a possible association between PE and PD	A positive association was found between PE and PD (OR 2.17, 95% CI 1.38–3.41, P=0.0008)<br>A high and significant heterogeneity was found ($\chi2$=62.42, P<0.00001, I2=75%)<br>In most cases, subgroup analysis had low power to detect significant differences between PE and non- PE groups
Desai [73]	2015	Case-control study	N=1240, PP (48h after delivery) among them non-preeclamptic women	To evaluate the association between maternal PD and PE	Maternal PD (OR 19.8) was associated with PE<br>Maternal PD remained associated with PE after matching for primiparity, which was another significant confounding factor (OR 9.33).

			(n=1120) and preeclamptic women (n=120)		
Huang [74]	2014	Meta-analysis	N/A	To evaluate the association between maternal PD and the risk of PE	P (<32 weeks of gestation) with PD had a 3.69-fold higher risk of developing PE compared with the one without PD (OR=3.69; 95% CI=2.58-5.27)<br>PD within 48 h prior to delivery was associated with a 2.68-fold higher risk of PE (OR=2.68; 95% CI=1.39-5.18)<br>P with PD within 5 days after delivery had a 2.22-fold higher risk of PE than P without PD (OR=2.22; 95% CI=1.16-4.27)
Popova [75]	2013	Literature review	N/A	To analyze microbiology of PD	*P. gingivalis, T. denticola* and *T. forsythia* form a consortium in the subgingival biofilm and are regarded as the principal periodontopathogenic bacteria<br>*A. actinomycetemcomitans, F. nucleatum, P. intermedia, C. rectus, P. migros, E. corrodens* are implicated in the PD process
Fox [76]	2019	Literature review	N/A	To review the latest evidence base and guideline updates surrounding the diagnosis, management, and fetal surveillance in PE, as well as its increasingly recognized role as an independent cardiovascular risk factor for the offspring	PE is a major cause of maternal morbidity and is associated with adverse fetal outcomes including intra-uterine growth restriction, preterm birth, placental abruption, fetal distress, and fetal death in utero<br>Management of the fetus in PE involves timely delivery and prevention of adverse effects of prematurity with antenatal corticosteroids and/or magnesium sulphate depending on gestation<br>PE has long-term adverse effects on the offspring
Beckers [77]	2020	Literature review	N/A	To investigate the role of the microbiome in the developing placenta and its potential contribution to PE	*Lactobacillus* (*Firmicutes*) is thought to play a major role in preventing overgrowth of pathogenic bacteria<br>*B. cereus, Listeria, Salmonella, Escherichia, K. pneumonia, Anoxybacillus, Variovorax, Prevotella, Porphyromonas*, and *Dialister* were found in placental samples from mothers with PE<br>High body mass index has been linked with PE
Aagaard [78]	2014	Case-cohort study	N=320	To characterize the microbiome of the placenta	The placental microbiome is composed of nonpathogenic commensal microbiota from the *Firmicutes, Tenericutes, Proteobacteria, Bacteroidetes*, and *Fusobacteria* phyla<br>The placental microbiome profiles were most akin (Bray-Curtis dissimilarity <0.3) to the human oral microbiome
Amarasekara [79]	2015	Case-control study	N=110, P with PE (n=55), P normotensive (n=55)	To detect, identify, quantify and compare the bacteria present in the placental tissues of women with PE with that of normotensive P	12.7% of placental tissue samples from P with PE were PCR-positive whereas all the placental samples from P normotensive were negative<br>The placental tissue samples from P with PE included *B. cereus*, *Listeria*, *Salmonella*, *Escherichia* (all of which are usually associated with gastrointestinal infection); *K. pneumonia* and *Anoxybacillus* (both of which are usually associated with respiratory tract infections); and *Variovorax, Prevotella, Porphyromonas,* and *Dialister* (all of which are usually associated with periodontitis)

Barak [80]	2007	Case-control study	N=30, P with PE (n=16), P without PE (n=14)	To explore the possibility that periopathogenic bacteria may translocate into the placental tissues of women with PE	50% of placenta specimens were positive for one or more periopathogenic bacteria in the PE group, compared to 14.3% from controls<br>Bacterial counts were statistically significantly higher in the PE group for all of the periopathogenic bacteria examined<br>All of the target periopathogenic bacteria were found in the PE group, only three (P. *gingivalis*, *T. forsythensis*, and *T. denticola*) were found in the control group
Han [81]	2011	Literature review	N/A	To review the current status of epidemiological, mechanistic, and intervention studies on oral health and adverse pregnancy outcomes	Positive association between PD and adverse pregnancy outcome<br>Two non-mutually exclusive hypotheses: (i) PD affect the maternal and fetal immune responses systemically, leading to premature labor, (ii) oral bacteria may translocate directly into the pregnant uterus, causing localized inflammation and adverse pregnancy outcome in the presence or absence of clinical PD
Andrukhov, [82]	2011	Case-control study	N=62, PD (n=37), PD healthy (n=25)	To investigate the levels of interferon (IFN)-γ, tumor necrosis factor (TNF)-α, and interleukin (IL)-2, -4, -5, and -10 in the serum of patients with PD in relation to the bacterial load in the dental plaques	Serum levels of IFN-g, TNF-a, andIL-10 were significantly increased, whereas those of IL-2 were significantly decreased in patients with PD compared to healthy<br>Increased serum levels of IFN-g and TNF-a in patients with PD were associated with the enhanced dental plaque load with *A. actinomycetemcomitans* and *P.gingivalis*, respectively<br>*A. actinomycetemcomitans* and *T. denticola* was associated with an increased population of CD3-/CD16+ and CD3+/CD8+ cells, respectively
Parthiban [83]	2018	Case-control study	N=50, P with PE (n=25), P normotensive (n=25)	To determine the association between the presence of specific periodontal pathogens, Toll-like receptor-4 (TLR-4), and nuclear factor-κB (NF-κB) expression in the placental tissues of pre-eclamptic women	The subgingival plaque samples of PE women showed significantly higher frequencies of *P. intermedia*<br>In the placental samples, *P. gingivalis, P. intermedia,* and the expression of TLR-4 and NF-κB were found to be at significantly higher levels compared to normotensive P<br>TLR-4 was significantly influenced by the presence of *P. gingivalis* and *P. intermedia*<br>NF-κB expression was influenced by the presence of *P. intermedia* in the placental tissues of PE women
Cleys [84]	2015	Observational study	N=3, P (n=3, 1st trimester)	To investigate the role of testosterone in placental gene expression, and focused on androgen receptor	In human first trimester placental samples, Androgen receptor complexes with histone lysine demethylases KDM1A and KDM4D immunolocalized to the syncytiotrophoblast, with nuclear KDM1A and KDM4D immunostaining also present in the villous stroma placental androgen signaling, possibly through Androgen receptor-KDM complex recruitment to androgen response elements, regulates placental VEGFA expression
Khong [86]	2011	Literature review	N/A	To analyze defective deep placentation	Defective deep placentation is characterized by defective remodeling of the utero-placental arteries<br>Under certain conditions, it is also characterized by the presence of arterial lesions, such as acute atherosis and the persistence of endovascular trophoblast

					Defective deep placentation is lied to abnormalities of the endometrium and inner myometrium before or during the early stages of placentation
Brosens [87]	2011	Literature review	N/A	To evaluate the biopsy technique to summarize the salient types of defective deep placentation and propose criteria for the classification of defective deep placentation into 3 types	Defective deep placentation is associated with a spectrum of obstetrical syndromes that included preeclampsia<br>These disorders of deep placentation are characterized by: (i) the degree of restriction of physiologic transformation of the spiral arteries and (ii) the presence of arterial lesions in the junctional zone myometrium of the placental bed
Vigliani [88]	2014	Literature review	N/A	To describe how the human placenta might be infected by microorganisms via the hematogenous route	Many blood-borne pathogens (*Listeria monocytogenes* or *Cytomegalovirus*…) might arrive at the placenta via transportation inside of maternal leukocytes that enter the decidua in early pregnancy<br>Extravillous trophoblasts could get infected in the decidua and spread infection to other layers in the placenta
Chaparro [89]	2013	Case-control study	N=58, Normotensive healthy P (n=33), P with hypertension (n=25)	To explore the associations between the presence of periodontal pathogens and the expression of TLR-2 and TLR-4 in the placental tissue of P with hypertensive disorders compared to the placentas of healthy normotensive P	A significant increase was observed in the expression of TLR-2 in the placentas of patients with hypertensive disorders<br>An association between the presence of *T. denticola* and *P. gingivalis* in placental tissues and hypertensive disorders was observed
Swati [90]	2012	Case-control study	N=20, Normotensive healthy P (n=10), P with hypertension (n=10)	To establish causal relationship between PD and PE<br>To find out causal circumstantial evidence by isolating specific periodontal pathogens in oral and placental samples	Periodontal pathogens (*P. gingivalis*, *F. nucleatum*, *T. denticola*, *P. intermedia* and *A. actinomycetemcomitans*) were found to be high in the group with hypertension than the controls<br>*P gingivalis* was found in all the samples from subgingival plaque and placenta, irrespective of the PD status.
Vanterpool [91]	2016	Case-control study	N=134, Preterm (n=97, ≤ 32 weeks gestation), Term (n=35)	To determine if the location of *P gingivalis* within placental and/or umbilical cord sections was associated with a specific delivery diagnosis at preterm delivery	*P gingivalis* within the placenta was significantly associated with shorter gestation lengths and delivery via caesarean section but not with histological chorioamnionitis or preeclampsia
Reyes [92]	2018	Literature review	N/A	To provide an overview of past and current research on *P. gingivalis* that addresses some of the controversies concerning the role of this organism in the pathogenesis of adverse pregnancy outcome	A combination of preexisting periodontal disease and *P. gingivalis* placental colonization would increase the risk of APO in part by inducing a TH17/Treg cell imbalance<br>*P. gingivalis* will be a vascular pathogen of the placental bed
